# Self-perceived attitudes toward interprofessional collaboration and interprofessional education among different health care professionals in pediatrics

**DOI:** 10.3205/zma001016

**Published:** 2016-04-29

**Authors:** Sebastian Felix Nepomuk Bode, Marianne Giesler, Andrea Heinzmann, Marcus Krüger, Christine Straub

**Affiliations:** 1 University Hospital Freiburg, Center for Pediatrics and Adolescent Medicine, Freiburg, Germany; 2 University Hospital Freiburg, Competency Centre for Evaluation in Medicine, Baden-Württemberg, Freiburg, Germany

**Keywords:** Interprofessional education, interprofessional collaboration, evaluation, pediatrics

## Abstract

Interprofessional education (IPE) is the basis for interprofessional collaboration (IPC) in health care systems. It has beneficial effects for both patients and health care professionals. IPC is paramount for adequate care of patients and their families, especially in pediatrics. To determine the attitudes of medical doctors (n=121), nurses (n=15), psychologists (n=14), and social workers (n=19) toward IPE and IPC in a tertiary pediatric university teaching hospital, as well as the inpatient and outpatient settings in pediatrics, we developed a questionnaire with 21 items in four categories based on established questionnaires. All participants worked as part of interprofessional teams, and the overwhelming majority valued IPC highly. Most competencies important for IPC were acquired on the job. There was a substantial lack of interprofessional education, especially for medical doctors and psychologists. IPE still needs to be established as part of the undergraduate curriculum at German universities.

## Zusammenfassung

Interprofessionelle Ausbildung (IPE) ist die Grundlage für interprofessionelle Zusammenarbeit (IPC) in Gesundheitssystemen. Sie hat sowohl auf die PatientInnen als auch auf die Angehörigen der Gesundheitsberufe positive Auswirkungen. Die IPC ist, insbesondere in der Pädiatrie, für eine angemessene Versorgung der PatientInnen und ihrer Familien von großer Bedeutung. Wir haben auf Grundlage etablierter Fragebögen einen Fragebogen mit 21 Fragen in 4 Kategorien entworfen, um die Einstellungen von ÄrztInnen (n=121), Pflegefachkräften (n=15), PsychologInnen (n=14) und SozialarbeiterInnen (n=19) in Bezug auf deren interprofessionelle Ausbildung und Zusammenarbeit in einem tertiären universitären pädiatrischen Zentrum, sowohl im Kontext der stationären als auch der ambulanten pädiatrischen Versorgung, zu untersuchen. Alle TeilnehmerInnen haben in interprofessionellen Teams gearbeitet und eine große Mehrzahl bewertet die IPC positiv. Die meisten Kompetenzen, die für die IPC von Bedeutung sind, wurden im Laufe der täglichen Arbeit erworben. Es bestand jedoch ein Mangel an interprofessioneller Ausbildung, insbesondere bei ÄrztInnen und PsychologInnen. Die interprofessionelle Ausbildung muss als Teil der Curricula der grundständigen Studiengänge an deutschen Universitäten etabliert werden.

## 1. Introduction

Interprofessional education (IPE), defined as students from two or more professions learning together, about, and from each other, is a necessity for successful interprofessional collaboration (IPC) [1], [2], [3], [4]. The ability to work as part of an interprofessional team or even lead an interprofessional team is expected of university graduates soon after they start their career in the health care system, but so far interprofessional learning has not been established in the standard curriculum of German universities. The CanMeds role model strongly emphasizes the “collaborator” as a learning objective in medical education and has been adopted by the education systems in different countries, such as Switzerland, and by some universities in Sweden and the United Kingdom [5], [6], [7], [8]. IPE is highlighted in the recently adopted German National Competency-based Learning Objectives Catalogue in Medicine (NKLM) [9], [http://www.nklm.de].

The WHO *Framework for Action on Interprofessional Education and Collaborative Practice* states that IPE improves both IPC and health care outcomes [1], especially in terms of shorter hospital stays for patients, less medication use, and greater appreciation of different health care professionals [10], [11], [12]. Two Cochrane reviews show that IPE and IPC do have positive effects, but that these cannot be generalized for behavioral changes in healthcare professionals or patient outcomes [13], [14].

Especially in the specialized care of pediatric patients (i.e. developmental aspects, family surroundings and social care), IPC is part of the daily routine work and crucial to adequate and economic care for patients and their families [15], [16]. 

Insufficient data exist on where and when different health care professionals acquire competencies essential for IPC. 

## 2. Aim

The aim of this study is to evaluate the attitudes of different health care professionals working in pediatrics toward IPE and IPC. We wanted to determine which competencies health care professionals acquired and whether those competencies were learned during undergraduate training or while performing day-to-day clinical work.

## 3. Methods

We reviewed different established tools that evaluate IPC, including the Readiness for Interprofessional Learning Scale (RIPLS) [17], the Generic Role Perception Questionnaire (GRPC) [18], the Interdisciplinary Education Perception Scale (IEPS) [19], and the Index of Interdisciplinary Collaboration (IIC) [20], [21]. Not all of these questionnaires have been validated in German. Some were too time-consuming due to a great number of questions, and not one questionnaire addressed all the questions we were aiming at. Consequently, we developed a questionnaire in German based upon the published questionnaires. We created a questionnaire comprising 21 items divided into four subcategories: interprofessional collaboration, interprofessional education, attitudes toward interprofessional collaboration in routine work, and visions for IPC and IPE, as well as six items on demographic data (see attachment 1 ). Possible answers were graded on 5 to 6-point Likert scales (for details please refer to the attachment 1 ). Two open-ended questions were asked concerning the future of IPC and IPE to supplement the quantitative part of the questionnaire with qualitative data. A main focus of the questionnaire was the evaluation of competencies important for IPC and IPE.

The questionnaire was peer-reviewed and approved by colleagues with a master’s degree in medical education and the Baden-Württemberg Competency Centre for Evaluation in Medicine in Freiburg, Germany.

All attendees of the 2014 meeting of the Working Group for child protection in medicine (AG-KiM) in Freiburg, Germany, were asked to fill out the questionnaire, as were all medical staff at the Freiburg Centre for Pediatrics and Adolescent Medicine (ZKJ), a tertiary university teaching hospital. These two groups did not overlap. The AG-KiM members work nationwide in Germany in inpatient and outpatient pediatric settings. The questionnaire was provided in electronic form (Questback GmbH. Published 2015. EFS survey, version 10.5. Cologne) at the ZKJ and in printed form to the AG-KiM, the latter was then entered into a joint electronic database.

## 4. Statistics

Statistical analysis was performed with IBM SPSS Statistics for Windows, (Version 22.0. Armonk, NY: IBM Corp.). Paired samples t-tests, Kruskal Wallis tests, and one-way ANOVAS were used to determine significant differences, and Bonferroni correction for multiple testing was applied where appropriate. Qualitative evaluation of the open-ended responses was performed as deductive and inductive qualitative content analysis after Mayring [22]. 

## 5. Results

### 5.1. Study population

A total of 168 completed questionnaires were returned. Of these, 116 participants were female, 48 were male, and four did not indicate their gender. A total of 121 medical doctors participated: fifty in the AG-KiM (return rate 50.5%) and 71 at the ZKJ (return rate 59.2%). Forty-seven participants who were not medical doctors included 15 nursing staff, 14 psychologists, 19 social workers, two teachers and five others (radiology assistant, doctor's assistant, psychotherapist, two unspecified). The eight participants indicating a dual education were allocated according to their highest academic degree. Only those groups with more than 10 participants were included in further analysis.

Of the medical doctors, 74 (61.1%) were board–certified pediatricians, 34 had additional subspecialty certification in pediatrics. 

Twenty-eight (23.1%) physicians had ≤ four years of professional experience, 40 (33.1%) had four-10 years of work experience, and 53 (43.8%) had ≥ 10 years of work experience. The other health care professionals generally had more work experience, with 68.4% (n=13) social workers, 80% (n=12) nurses and 92.9% (n=13) psychologists having ≥ 10 years of work experience.

#### 5.2. Interprofessional collaboration

**Figure 1:**
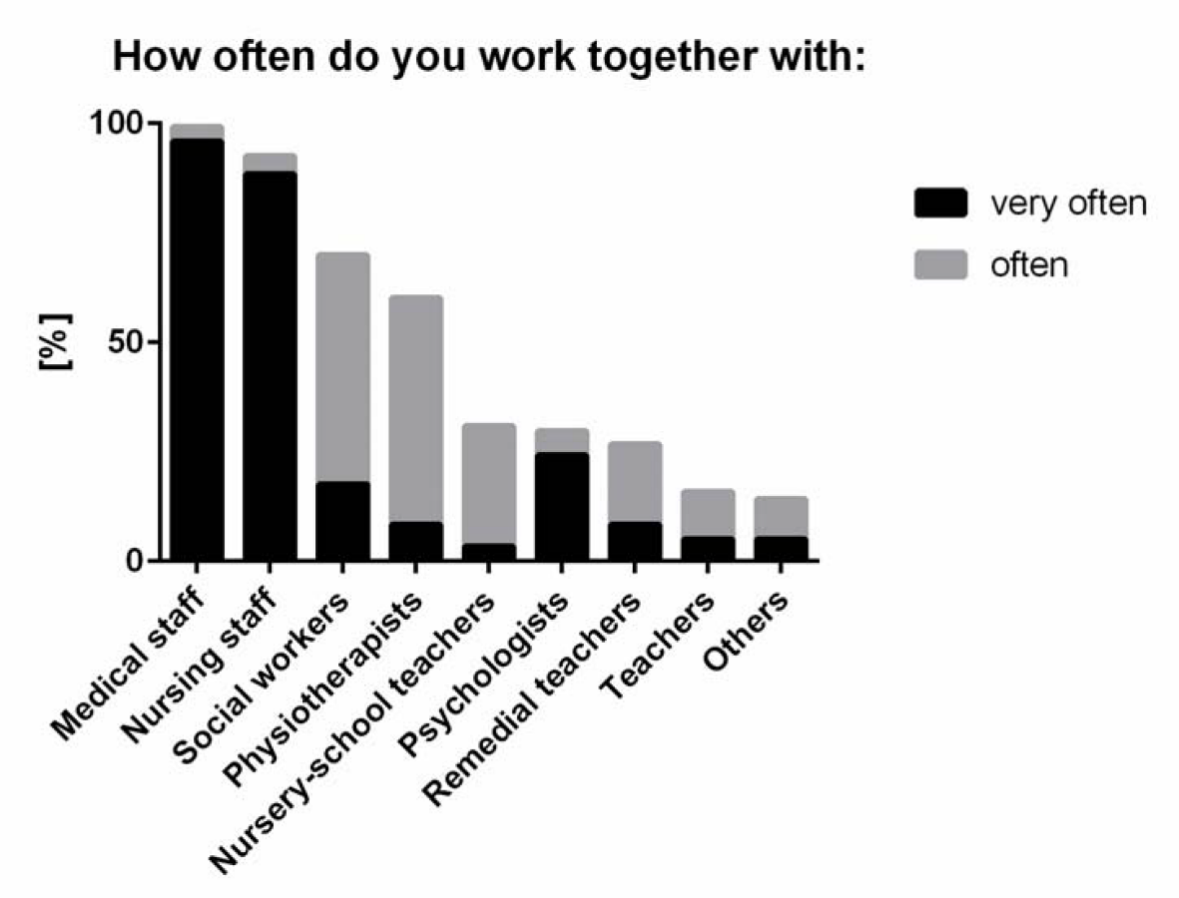
Frequency of medical doctors' collaboration with different health care professions. Participants were asked to answer: “Which professional groups are you working with, and how frequently, in your routine work? Please provide information for each professional group.” The ranking was performed by pooling “very often” and “often.”

IPC was reported to be part of the daily routine by almost all medical doctors. A total of 120 physicians (99.2%) worked together with other physicians frequently or very frequently. Frequent or very frequent collaboration with nursing staff was reported by 111 (91.7%), with social workers by 85 (70.2%), with physiotherapists by 73 (60.3%), with nursery-school teachers by 37 (30.5%), with psychologists by 36 (29.7%), with remedial teachers by 32 (26.4%), with teachers by 19 (15.7%), and with other health care professionals (e.g. midwives) by 17 (14%) of the physicians (see figure 1 (Fig. 1)).

In the AG-KiM, 92.9% of psychologists worked together with social workers, and 100% of social workers worked at least frequently with psychologists, whereas only 78.6% of psychologists and 78.9% of social workers worked frequently or very frequently together with nursing staff. 

**Figure 2:**
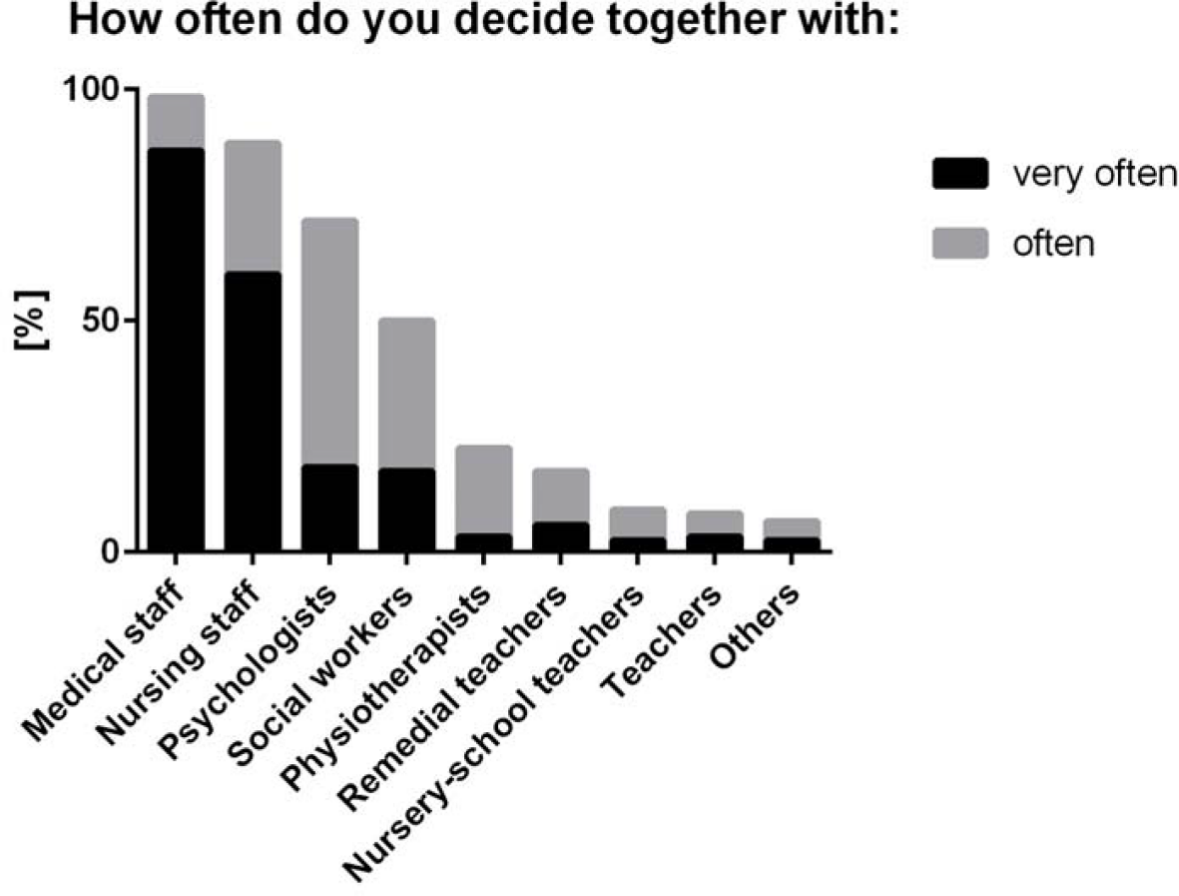
Frequency of medical doctors' decision-making with different health care professions. Participants were asked to answer: “How often do you make decisions in an interprofessional team? Please provide information for each professional group.” The ranking was performed by pooling “very often” and “often.”

Very frequent or frequent team decisions with other physicians were reported by 119 (98.3%), with nursing staff by 107 (88.4%), with psychologists by 87 (71.9%), with social workers by 61 (50.4%), with physiotherapists by 27 (22.3%), with remedial teachers by 21 (17.3%), with nursery-school teachers by 11 (9.1%), with teachers by 10 (8.3%), and with other health care professionals by 8 (6.6%) of the physicians (see figure 2 (Fig. 2)). Medical doctors at the ZKJ (M=1.37, SD±0.54) did include nursing staff significantly more frequently in interdisciplinary decisions than did physicians in the AG-KiM (M=1.82, SD±1.35) (p=.032). Psychologists did not value nursing staff as highly as other health care professionals in interprofessional decisions (“nursing staff” (M=1.43, SD±0.76) vs. “other health care professions” (M=1.14, SD±0.36): p=.0203). 

**Figure 3:**
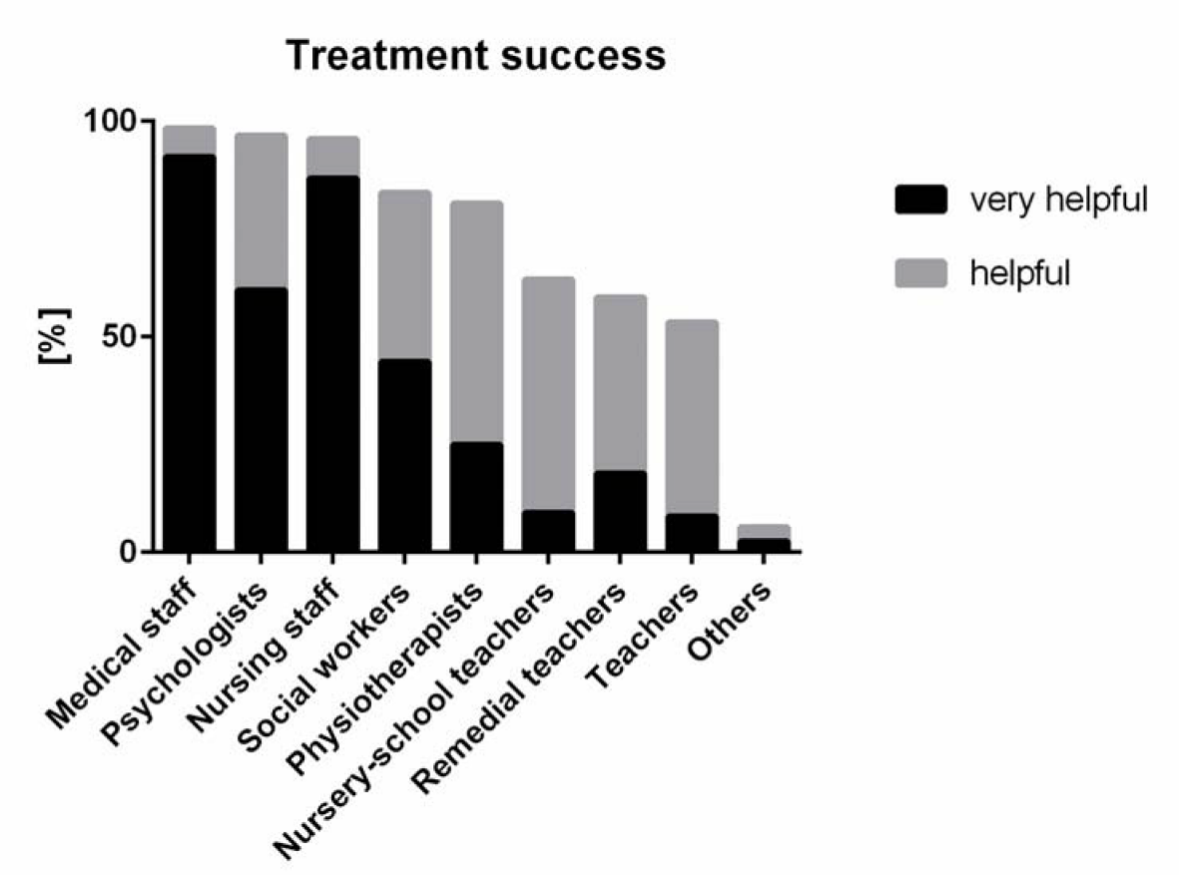
Frequency of medical doctors' decision-making with different health care professions. Participants were asked to answer: “For successful treatment of the patient/client the interprofessional collaboration with the following professional groups is.... Please provide information for each professional group.” The ranking was performed by pooling “very often” and “often.”

Of all participants, 90.9% (153) stated that IPC in routine clinical work was “very helpful;” all but one of those remaining rated it as “helpful”. The collaboration with different health care professions was deemed important for treatment success (see figure 3 (Fig. 3)). IPC with other physicians was stated to be very helpful or helpful by 119 (98.3%), with psychologists by 117 (96.7%), with nursing staff by 116 (95.9%), with social workers by 100 (82.6%), with physiotherapists by 98 (80.9%), with nursery-school teachers by 77 (63.6%), with remedial teachers by 72 (59.5%), and with others by 7 (5.8%) of the participating physicians (see figure 3 (Fig. 3)). No demographic factors (sex, work experience, affiliation to ZKJ or AG-KiM) or profession accounted for any differences.

#### 5.3. Interprofessional education

Neither work experience, sex, nor affiliation with the AG-KiM or ZKJ had any influence on how much opportunity medical doctors had had to participate in interprofessional education during undergraduate training. Surprisingly, significantly more options for interprofessional learning on the job (M=3.77, SD±1.15) were reported compared to undergraduate training (M=4.72, SD±0.99, p<.0001). The same held true for the acquisition of competencies necessary for IPC, for example assessment of one's professional role, definition of other roles, conflict management, respect for other professions, error management, medical terminology, and interprofessional collaboration in general which were reported to have been acquired significantly more often during work than during undergraduate training (see table 1 (Tab. 1)). Significantly more medical doctors indicated not having acquired any interprofessional competencies at all during undergraduate training (see table 1 (Tab. 1)). By trend, psychologists did not acquire any of the competencies during undergraduate training but only during work, whereas social workers mainly learned respect for other health care professionals, adequate terminology, and interprofessional collaboration in general during work. Nurses acquired all competencies apart from conflict management during undergraduate training. The self-assessed level of competence did not differ significantly between the professions.

#### 5.4. Day-to-day interprofessional collaboration

In general, all participants demonstrated a high level of awareness of interprofessional collaboration. Work experience, affiliation with the AG-KiM or ZKJ, sex, or profession did not have any influence on this awareness. 

Competencies acquired during undergraduate training and, more importantly, during clinical work did lead to significantly higher levels of self-assessment:

Medical doctors at the ZKJ who had stated having learned more error culture during undergraduate training (M=1.59, SD±0.76) thought it significantly more important to include all relevant professions in interprofessional care than did other colleagues (M=1.8, SD±0.86, p=.044). 

Role conflicts were less troublesome for those who were able to notice role conflicts in the first place (M=2.86, SD±0.94) compared to those who did not notice role conflicts as well (M=3.21, SD 0.76, p=.032).

Medical doctors who reported that they had learned to respect other health professions during their work (M=2.37, SD±0.91) agreed significantly more strongly that the work of other health professions is not valued enough in routine work than those who had not learned to respect others (M=1.88, SD±1.03, p=.019). Those, and those who were able to identify their own role (M=2.23, SD±0.95), did state significantly less frequently that the leader of an interprofessional team needed to be a medical doctor compared to those who did not state having acquired the particular competencies (M=2.85, SD±1.21, p=.039).

Hierarchy was rated as less important by physicians with more than 10 years of professional experience (M=2.2, SD±0.71) in comparison to those with less than six years of professional experience (M=1.74, SD±0.81, p=.02). Communication was rated less important by those with a moderate amount of work experience (six-10 years) (M=1.33, SD±0.53) compared to less experienced physicians (M=1.04, SD±0.2, p=.029). 

Between participating nurses, psychologists and social workers, no significant correlations could be determined concerning the acquisition of competencies and higher self-assessment or of the ability to work interprofessionally. This might be due to the relatively low numbers of participants in these groups.

#### 5.5. Visions

The majority of the participants, 75.9% (n=128), rated the future importance of IPE as “very relevant” or “relevant.” Significantly more, namely 94.6% (n=159), rated the future importance of IPC as “very relevant” or “relevant” (p<.001). Interestingly, female doctors (M=1.92, SD±0.81) rated IPE significantly more frequently as “very relevant” than their male counterparts (M=2.31, SD±0.99, p=.036). Young physicians (< six years of work experience) at the ZKJ (M=1.05, SD±0.23) indicated a higher importance of IPC than their more experienced colleagues (M=1.43, SD±0.64, p=.013). There were no significant differences between professions.

#### 5.6. Qualitative evaluation

Through deductive and inductive context analysis based on Mayring [22], we were able to identify four core competencies that the participants rated as being most important for the future of IPE and IPC, these being professional expertise, methodical expertise, social expertise, and personal competence. 

Professional expertise was mentioned as the basis of future IPE and IPC. Participants suggested using functioning structures in IPC (e.g. ethics committees, child protection services) as examples for IPE and establishing interprofessional lectures and courses. Methodical expertise (e.g. conflict management) was deemed important to cope with challenging situations; social competence to enable interaction in a social environment. Personal competence is comprised of the abilities to self-reflect and anticipate the consequences of actions undertaken in IPC. 

Structural changes, for example in the clinical hierarchy or allotting more time for IPE and IPC, along with the possibility of interprofessional courses during undergraduate and postgraduate work, were also highlighted as essential.

## 6. Discussion

This is the first larger study that evaluates interprofessional education (IPE) and interprofessional collaboration (IPC) in the pediatric care setting. 

The overwhelming majority of participants did show a high level of awareness for IPC and ranked IPC as important for the clinical care of patients. Only for single items were we able to determine significant differences in attitudes that could be attributed to work experience, age, sex, working in a pediatric inpatient or outpatient setting. For example, less experienced medical doctors rated the importance of IPC as being higher than their more experienced peers did, and female medical doctors valued IPE more highly than their male colleagues in general, different health care professionals in pediatrics valued the role of IPE and IPC.

### 6.1. Interprofessional Collaboration

As expected, medical doctors most frequently worked together and made joint decisions with nursing stuff, more so in the inpatient pediatric setting. This is not surprising as pediatricians in ambulatory care in Germany traditionally do not work with qualified nurses but rather with physician's assistants. At the ZKJ, psychologists seem to play an important role in the interprofessional teams, so it is not surprising that this study showed a high level of collaboration with this profession. In the AG-KiM, psychologists and social workers did work significantly more with each other than with any other health care professionals, which is typical for many pediatric in- and outpatient settings in Germany.

We were also able to show that competencies acquired during undergraduate training or during the professional career led to a higher appraisal of different professional groups and more collaboration with other professions in day-to-day work, something that is corroborated by the literature [13], [14]. The core competencies stated by participants, either by checking items or as commentary, are congruent with the competencies proposed by the expert panels on interprofessional collaboration [4], [7].

#### 6.2. Interprofessional education

When comparing the results of the different professional groups, it must be kept in mind that the non-medical participants had their professional education mostly more than 10 years ago. The most important finding was the absence of structured IPE during undergraduate training and that significantly more participants acquired skills necessary for IPC in the course of their work. This correlates with findings concerning other professional groups. For example, only 43% of physical therapists (PT) and 50% of occupational therapists (OT) in Canada reported having received sufficient IPE [23]. Both our data and the literature may hint at a need for greater promotion of IPE as a basis on which IPC can gain support to initiate IPE programs, especially in medical schools and during undergraduate psychology, PT, and OT training. It will be interesting to see how today's students in healthcare-related professions rate their acquisition of IPC skills. Social workers and especially nurses seem to acquire more competencies important for IPC during undergraduate training.

Interestingly, the participants rated the future importance of IPC as being higher than that of IPE. This does put an additional strain on junior staff in health care who will have to cope with their new role, continue to gain knowledge in their specialties, and at the same time focus on how to improve their interprofessional competencies [9], [24]. 

In Germany, the National Competency-based Catalogue of Learning Objectives in Medicine (NKLM) from 2015 does put emphasis on the acquisition of competencies, including those essential to IPC [9], [http://www.nklm.de]. The implementation of the NKLM into the curriculum of German medical schools should enable medical students and junior medical doctors to fulfill their roles in the interprofessional team more quickly and efficiently, to the benefit of patients and treatment outcomes. Initial projects to implement IPE in university courses in Germany include the bachelor's degree in interprofessional health care studies at Heidelberg and the program entitled, *Longitudinaler Strang Interprofessionalität* (Longitudinal Focus on Interprofessionalism), at Freiburg which include other health care professionals as well [25], [26]. It would be beneficial to analyze how IPE takes place and what exactly is learned in IPE during professional practice, as well as whether or not a theory of social learning could explain these aspects of IPE.

#### 6.3. Limitations and outlook

This study has some limitations in the field of IPE and IPC as reported in other studies [13], namely the small number of participants and the heterogeneous study cohort with a predominance of female participants. Our project evaluates the attitudes toward IPE and IPC held by health care professionals working in pediatrics, as well as the acquisition of competencies important for IPE and IPC. Of all participants in our study, 72% were medical doctors making the interpretation of these results difficult. Further evaluation of the questionnaire is planned. An interesting long-term project will be evaluating the acquisition of competencies by future medical students as the NKLM [9], [http://www.nklm.de] is implemented in the coming years.

## 7. Conclusions

Interprofessional collaboration is an essential part of routine work in pediatrics and is valued highly by different health care professionals who seem to acquire interprofessional skills mostly during clinical work and in direct interaction with other professionals. There is a substantial lack of interprofessional education at German medical schools and universities that should be addressed urgently; initial projects are underway. 

## Acknowledgements

We wish to thank all those who participated in the survey and the organizers of the 2014 meeting of the Working Group for child protection (AG-KIM) in Freiburg, Germany, for the opportunity to recruit participants for our study. 

## Competing interests

The authors declare that they have no competing interests.

## Supplementary Material

Questionnaire (translated from the original German version)

## Figures and Tables

**Table 1:**
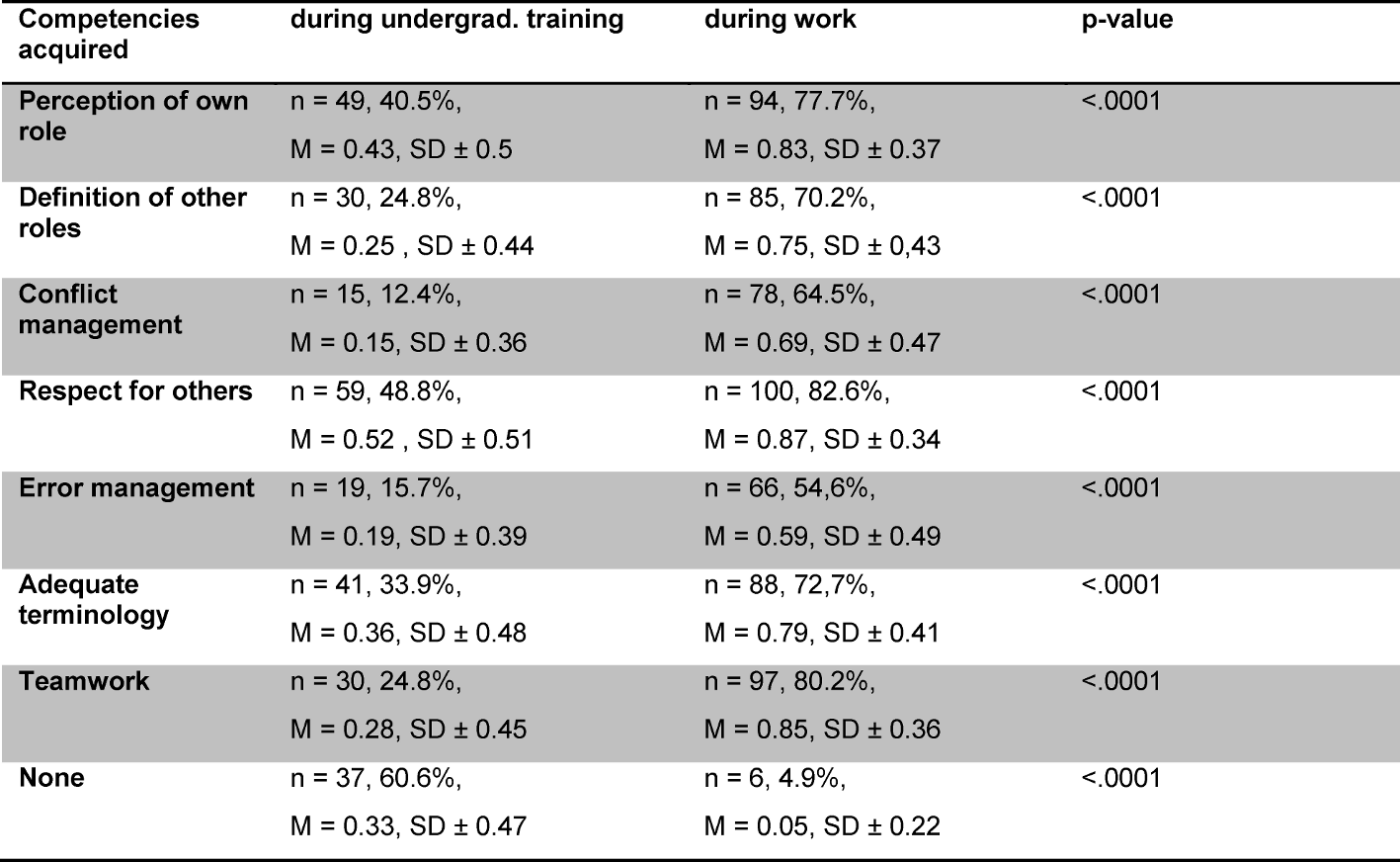
Competencies in IPC acquired by medical doctors during undergraduate studies and during professional work. Each row represents a different competence; n=number of participants stating they acquired the relevant competence during undergraduate studies or on the job, followed by percentage of the 121 participating physicians. M=mean, SD=standard deviation.
