# Supplementary material for: Self-perceived attitudes toward interprofessional collaboration and interprofessional education among different health care professionals in pediatrics
Source: GMS J Med Educ. 2016 Apr 29;33(2):Doc17. doi: 10.3205/zma001016 (PMC4895846; doi:10.3205/zma001016)
Supplement: Questionnaire (translated from the original German version) [file JME-33-17-s-001.pdf]

Appendix

Appendix I: Questionnaire (translated from the original German version)

1) Which professional groups are you working with, and how frequently, in your routine work?

Please provide information for each professional group. If necessary, enter other professions in the free text box.

	very frequently	frequently	infrequently	rarely	never
Medical doctors	□	□	□	□	□
Nurses	□	□	□	□	□
Psychologists	□	□	□	□	□
Physiotherapists	□	□	□	□	□
Nursery school teachers	□	□	□	□	□
Hospital school teachers	□	□	□	□	□
Social workers	□	□	□	□	□
Remedy teachers	□	□	□	□	□
others:	□	□	□	□	□

2) How often do you make decisions in the interprofessional team?

Please provide information for each professional group. If necessary, enter other professions in the free text box.

	very frequently	frequently	infrequently	rarely	never
Medical doctors	☐	☐	☐	☐	☐
Nurses	☐	☐	☐	☐	☐
Psychologists	☐	☐	☐	☐	☐
Physiotherapists	☐	☐	☐	☐	☐
Nursery school teachers	☐	☐	☐	☐	☐
Hospital school teachers	☐	☐	☐	☐	☐
Social workers	☐	☐	☐	☐	☐
Remedy teachers	☐	☐	☐	☐	☐
others:	☐	☐	☐	☐	☐

3) For successful treatment of the patient/client the interprofessional collaboration with the following professional groups is:

Please provide information for each professional group. If necessary, enter other professions in the free text box.

	very helpful	helpful	neither/nor	hindering	very hindering
Medical doctors	☐	☐	☐	☐	☐
Nurses	☐	☐	☐	☐	☐
Psychologists	☐	☐	☐	☐	☐

Physiotherapists	☐	☐	☐	☐	☐
Nursery school teachers	☐	☐	☐	☐	☐
teachers	☐	☐	☐	☐	☐
Social workers	☐	☐	☐	☐	☐
Remedy teachers	☐	☐	☐	☐	☐
others:	☐	☐	☐	☐	☐

4) For me personally, the interprofessional collaboration with other professional groups is:

very helpful	helpful	neither/nor	hindering	very hindering
☐	☐	☐	☐	☐

5) How many opportunities for interprofessional learning did you have during your studies / your undergraduate training?

Too many	many	enough	few	Very few	none
☐	☐	☐	☐	☐	☐

6) I have acquired the following competencies for interprofessional cooperation during my studies / my undergraduate training?

Please tick all items that apply; you can also add further items if needed.

- ◻ Perception of one's professional role
- ◻ Definition of others's professional roles
- ◻ Conflict management
- ◻ Respect for other professions
- ◻ Error management
- ◻ Talk with other professional groups in an appropriate technical language
- ◻ Initiate and maintain cooperation with other professional groups
- ◻ None of the above
- ◻ others:

7) How many opportunities for interprofessional learning do you have during your everyday work life?

Too many	many	enough	few	Very few	none
◻	◻	◻	◻	◻	◻

8) I have acquired the following competencies for interprofessional cooperation during my everyday work life?

Please tick all items that apply; you can also add further items if needed.

- ◻ Perception of one's professional role
- ◻ Definition of others's professional roles
- ◻ Conflict management
- ◻ Respect for other professions
- ◻ Error management

- ◻ Talk with other professional groups in an appropriate technical language
- ◻ Initiate and maintain cooperation with other professional groups
- ◻ None of the above
- ◻ others:

9) In (clinical) routine work, it is essential to involve all relevant professional groups in the decision-making process .

totally agree	Agree	neither/nor	disagree	strongly disagree	No opinion
◻	◻	◻	◻	◻	◻

10) I am able to differentiate the roles and responsibilities of different professional groups.

totally agree	Agree	neither/nor	disagree	strongly disagree	No opinion
◻	◻	◻	◻	◻	◻

11) I am able to consider information from other professional groups in an appropriate manner during history taking.

totally agree	Agree	neither/nor	disagree	strongly disagree	No opinion
◻	◻	◻	◻	◻	◻

12) The evaluation of interprofessional collaboration with other team members is part of my daily work.

totally agree	Agree	neither/nor	disagree	strongly disagree	No opinion
□	□	□	□	□	□

13) I am able to perceive role conflicts in interprofessional teams.

totally agree	Agree	neither/nor	disagree	strongly disagree	No opinion
□	□	□	□	□	□

14) Role conflicts in interprofessional teams are handled adequately in everyday work.

totally agree	Agree	neither/nor	disagree	strongly disagree	No opinion
□	□	□	□	□	□

15) The work of other professional groups is often not appreciated enough during everyday work.

totally agree	Agree	neither/nor	disagree	strongly disagree	No opinion
□	□	□	□	□	□

16) An interprofessional team should be led by a medical doctor.

totally agree	Agree	neither/nor	disagree	strongly disagree	No opinion
◻	◻	◻	◻	◻	◻

17) Which of the following conditions are important to you personally for a successful interprofessional collaboration?

If necessary, enter further conditions in the free text box.

	very important	important	neither /nor	insignificant	very insignificant
Knowledge about the health care system	◻	◻	◻	◻	◻
Interprofessional learning	◻	◻	◻	◻	◻
time	◻	◻	◻	◻	◻
Appropriate communication	◻	◻	◻	◻	◻
Flat hierarchy	◻	◻	◻	◻	◻
Knowledge of the roles and responsibilities of other professional groups	◻	◻	◻	◻	◻
Conflict management	◻	◻	◻	◻	◻
error culture	◻	◻	◻	◻	◻
patient orientation	◻	◻	◻	◻	◻

Economic facilities	☐	☐	☐	☐	☐
Others:	☐	☐	☐	☐	☐

18) How do you rate the importance of interprofessional education in medicine and medical professions in the coming years?

very relevant	relevant	somehow relevant	less relevant	not relevant	not at all relevant
☐	☐	☐	☐	☐	☐

19) How do you rate the importance of interprofessional collaboration in medicine and related fields in the coming years?

very relevant	relevant	somehow relevant	less relevant	not relevant	not at all relevant
☐	☐	☐	☐	☐	☐

20) What are your visions for the future of interprofessional education?

21) What are your visions for the future of interprofessional collaboration?

22) Please indicate your gender:

female	male
☐	☐

23) Please indicate how many years of work experience you have:

< 1 year	1-4 years	4-6 years	6-10 years	> 10 years
☐	☐	☐	☐	☐

24) Please indicate how many years of work experience in Pediatrics you have:

none	< 1 year	1-4 years	4-6 years	6-10 years	> 10 years
☐	☐	☐	☐	☐	☐

25) Please enter your professional education:

Please tick all that apply; you can also add further information.

☐	Nursing/nursing studies
☐	Physiotherapy
☐	Medicine
☐	Dentistry
☐	Psychology
☐	Social work

- ☐ Orthopedagogy
- ☐ Pedagogics
- ☐ Others:

Only for medical doctors:

26) Have you have completed a specialist training?

yes	no
☐	☐

27) Have you have completed a specialist training in Pediatrics?

yes	no
☐	☐

Thank you for your participation!
